# The mitochondrial genome of the orange-striped green sea anemone Diadumene lineata (Actiniaria: Diadumenidae): the first complete sequence in the family Diadumenidae

**DOI:** 10.1080/23802359.2019.1710594

**Published:** 2020-01-14

**Authors:** Haiyan Cong, Yixuan Lei, Lingming Kong

**Affiliations:** a Weihai Clinical Medical School, Cheeloo College of Medicine, Shandong University, Weihai, P.R. China;; b Marine College, Shandong University, Weihai, P.R. China

**Keywords:** *Diadumene lineata*, mitogenome, phylogeny

## Abstract

The complete mitogenome of the orange-striped green sea anemone (*Diadumene lineata*) has been sequenced and annotated for the first time. The total length of the mitogenome is 17,552 bp with an A+T content of 62.6%. Unlike typical metazoan mitogenome, this mitogenome include 14 protein-coding genes (13 energy pathway protein coding genes, and a heg gene), two tRNAs, two rRNAs, and 19 intergenic regions. The COX1 gene possesses a homing endonuclease gene. This circular genome contains two introns, one in ND5 and another in COX1.This sequence is the first sequenced complete mitogenome in Diadumenidae and provides fundamental data for exploring complicated evolutionary relationships in Actiniaria.

The *Diadumene lineata* belongs to Diadumenidae (Actiniaria), historically known as *Sagartia lineata*, *Sagartia luciae*, *Haliplanella luciae*, *Haliplanella lineata*, or *Diadumene luciae* (Hancock et al. 2017). It is morphologically distinctive from other anemones with distinct vertical stripes (Ruppert and Fox 1988). Diadumenidae is a monogeneric family comprising approximately 10 described species (Fautin 2013). The complete mitogenome of species in Diadumenidae has not been sequenced yet. Here, we first report the mitogenome of *Diadumene lineata* collected from the coastal rock of Jing Shui bay, Weihai, Shandong Province, P.R. China (E122°7′17.04″, N37°32′58.56″).The specimen and its DNA were stored in the Laboratory of Molecular Biology, Marine College, Shandong University (KC-ZTJHK-006). The complete mitogenome of the specimen was determined using Sanger sequencing. The mitogenome has been deposited in the NCBI GenBank under the accession number MH699974.

The total length of *D. lineata* mitogenome is 17,552 bp, bigger than *Nematostella* sp. (16,389), but smaller than most actiniarian species. The mitogenome contains 14 PCGs (ND1 ∼ 6, ND4L, COX1 ∼ 3, ATP6, ATP8, CYTB, and heg), two tRNA genes (tRNA-Trp and tRNA-Met), and two rRNA genes (12s ribosomal RNA and 16s ribosomal RNA). Unlike the typical metazoan mitogenome, the mitogenome of the sea anemone has intron interrupting ND5 and COX1 genes. The COX1 gene possesses a homing endonuclease gene (heg). All the 18 genes are transcribed from the heavy (H) strand. The A + T richness is 62.6%, similar to other actiniarian mitochondrial nucleotide composition (Foox et al. 2016). The GC skew of the whole mitogenome is 0.114 while the AT skew is −0.129, which means the mitogenomic contains more T than A, and more G than C. Similar favor of T and G was also found in other sequenced actiniarian mitogenomes (Zhang et al. 2017). Two introns were found in ND5 and COX1. The ND1 and ND3 genes locate in the ND5 intron and the heg gene cut the COX1 intron into two pieces. There is no overlap between genes but 19 intergenic spacers are observed in the mitogenome, ranging from 4 bp to 324 bp.

Nineteen species with complete 13 PCGs (excluding termination codons) are used in phylogenetic analyses, *Savalia savaglia* used as outgroup. Each PCG is aligned individually with codon-based multiple alignments using MUSCLE v3.8.31(Edgar 2004). The third codon position of PCGs is the most variable (Xia 1998), and likely experienced substitution saturation and consequently cause homoplasy in nucleotide gene data, so the third bases of each codon are excluded. Alignments of individual genes are then concatenated as a combined matrix. Maximum-likelihood (ML) tree are inferred using IQ-tree (Nguyen et al. 2015) using the models detected with ModelFinder (Kalyaanamoorthy et al. 2017), and node confidence was assessed with 1000 ultrafast bootstrap replicates. Our phylogenetic analysis strongly supports that *D. lineata* is sister to *Metridium senile*, and further confirms that Diadumenidae was closely related to members of Metridiidae (Figure 1) (Daly et al. 2008; Grajales and Rodríguez 2016).

**Figure 1.**
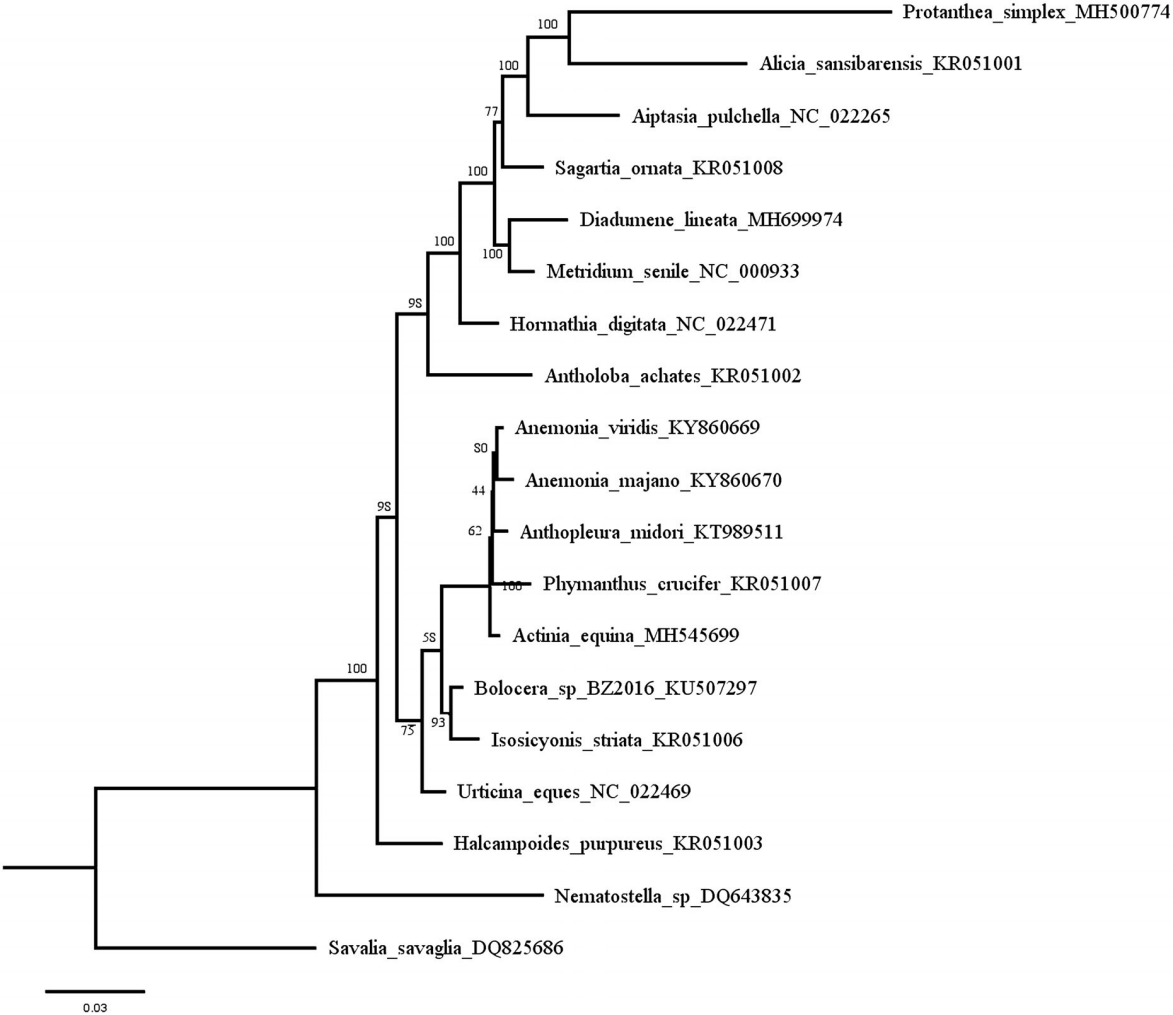
Maximum-likelihood tree of Actiniaria based on the combined dataset of first and second codon positions of 13 PCGs. Numbers above branches indicate maximum-likelihood bootstrap support values. The best model for this combined dataset was TPM3u + F+R3.

## References

[CIT0001] Daly M, Chaudhuri A, Gusmão L, Rodríguez E. 2008. Phylogenetic relationships among sea anemones (Cnidaria: Anthozoa: Actiniaria). Mol Phylogenet Evol. 48(1):292–301. 18413291 10.1016/j.ympev.2008.02.022

[CIT0002] Edgar R. C. 2004. MUSCLE: multiple sequence alignment with high accuracy and high throughput. Nucleic Acids Res. 32(5):1792–1797. 15034147 10.1093/nar/gkh340 PMC390337

[CIT0003] Fautin D. 2013. Hexacorallians of the World. [Internet resource]. [accessed 2019 November 28]. http://hercules.kgs.ku.edu/hexacoral/anemone2/index.cfm

[CIT0004] Foox J, Brugler M, Siddall M. E, Rodríguez E. 2016. Multiplexed pyrosequencing of nine sea anemone (Cnidaria: Anthozoa: Hexacorallia: Actiniaria) mitochondrial genomes. Mitochondrial DNA A. 27(4):2826–2832. 10.3109/19401736.2015.1053114 26104159

[CIT0005] Grajales A, Rodríguez E. 2016. Elucidating the evolutionary relationships of the Aiptasiidae, a widespread cnidarian–dinoflagellate model system (Cnidaria: Anthozoa: Actiniaria: Metridioidea). Mol Phylogenet Evol. 94:252–263. 26375331 10.1016/j.ympev.2015.09.004

[CIT0006] Hancock Z. B, Goeke J. A, Wicksten M. K. 2017. A sea anemone of many names: a review of the taxonomy and distribution of the invasive actiniarian *Diadumene lineata* (Diadumenidae), with records of its reappearance on the Texas coast. ZK. 706:1–15. 10.3897/zookeys.706.19848 PMC5674082 29118617

[CIT0007] Kalyaanamoorthy S, Minh B. Q, Wong T. K. F, Haeseler A, von Jermiin L. S. 2017. ModelFinder: fast model selection for accurate phylogenetic estimates. Nat Methods. 14(6):587–589. 28481363 10.1038/nmeth.4285 PMC5453245

[CIT0008] Nguyen L.-T, Schmidt H. A, von Haeseler A, Minh B. Q. 2015. IQ-TREE: a fast and effective stochastic algorithm for estimating maximum-likelihood phylogenies. Mol Biol Evol. 32(1):268–274. 25371430 10.1093/molbev/msu300 PMC4271533

[CIT0009] Ruppert E. E, Fox R. S. 1988. Seashore animals of the Southeast. Columbia: University of South Carolina Press.

[CIT0010] Xia X-H. 1998. The rate heterogeneity of nonsynonymous substitutions in mammalian mitochondrial genes. Mol Biol Evol. 15(3):336–344. 9501500 10.1093/oxfordjournals.molbev.a025930

[CIT0011] Zhang B, Zhang Y.-H, Wang X, Zhang H.-X, Lin Q. 2017. The mitochondrial genome of a sea anemone *Bolocera* sp. exhibits novel genetic structures potentially involved in adaptation to the deep-sea environment. Ecol Evol. 7(13):4951–4962. 28690821 10.1002/ece3.3067 PMC5496520

